# Growth Performance, Carcass Characteristics and Meat Quality of Organically Reared Broiler Chickens Depending on Sex

**DOI:** 10.3390/ani11113274

**Published:** 2021-11-16

**Authors:** Dorota Cygan-Szczegielniak, Joanna Bogucka

**Affiliations:** Department of Animal Physiology and Physiotherapy, Faculty of Animal Breeding and Biology, Bydgoszcz University of Science and Technology, Mazowiecka 28, 85-004 Bydgoszcz, Poland; bogucka@pbs.edu.pl

**Keywords:** organic chicken, sex, pectoral muscle, performance traits, meat quality, fatty acid profile, fiber diameter

## Abstract

**Simple Summary:**

Consumers are increasingly interested in the health and nutritional aspects of meat products, with the result that they are willing to pay more for meat products that have been produced naturally, taking into account high standards and animal welfare. Therefore, we decided to examine in a slightly wider perspective the muscles of Ross 308 chickens kept in an ecological system, taking into consideration an additional factor, i.e., sex. As sexual dimorphism is considered to be a factor in meat quality, we decided to examine this factor in our study. At the same time, we investigated the suitability of these fast-feathering broilers for ecological production. The aim of the study was to investigate the effect of sex on the growth performance, carcass traits, meat quality, fatty acid profile and histological traits of the pectoral muscles in organic Ross 308 broiler chickens. The suitability of these fast-growing broilers for organic production systems was also analyzed. As expected, the study confirmed the influence of sex on the analyzed parameters of the pectoral muscles in chickens reared in the organic system.

**Abstract:**

Given the growing interest of consumers in naturally produced meat, we decided to examine the muscles of Ross 308 broiler chickens kept in an ecological system, with the division into two research groups depending on sex. All the analyses were carried out using the appropriate methods recommended by the AOAC and in accordance with the Polish standards (PN), which are described in detail in the relevant section of the publication. The aim of the experiment was to investigate the effect of sex on the growth performance, carcass traits and meat quality, as well as the fatty acid profile and histological parameters, of the pectoral muscles from organic broiler chickens. A total of 60 one-day-old Ross 308 chickens (half males and half females) were divided into two groups, according to the sex, and reared under organic conditions (Org.) until 82 days of age (ten birds, i.e., five males and five females in each of three pens; replications for experimental groups). Compared with the female group, the male group had a higher final BW and carcass weight (*p* < 0.05). The males had a better growth and slaughter performance than the females. The meat quality traits and fatty acids content were also affected by sex. The meat from females showed a significantly higher (*p* < 0.05) protein, dry matter, fiber diameter and shear force and a significantly lower (*p* < 0.05) fat level than the male group. In this research, the suitability of these fast-growing broilers for natural and organic production systems has been researched with regard to the performance, meat quality and histological characteristics of the muscles.

## 1. Introduction

Meat products with high nutritional value and coming from organic systems with animal welfare in mind are becoming more and more popular among consumers. Therefore, in the USA, the EU and other regions of the world, this fact has contributed to a significant development of poultry meat production in less intensive systems [1]. Moreover, consumers in the European Union are showing an increased interest in poultry from alternative management systems, i.e., free-range and organic, which has resulted in a 14% increase in the production of poultry reared in these conditions [2]. However, organic animal production still remains limited compared to the total animal production in Europe and the European Union, ranging from 0.5 to 4% depending on the animal species [3]. The standards of organic poultry production are specified in Commission Regulation (EC) No. 889/2008 [4]. In this regulation, organic poultry production is defined as the production of slow-growing or fast-growing strains reared to a minimum age of 81 days. The housing conditions should provide a high level of animal welfare, and the animals should have access to pastures and receive feed coming from the local farm. The animals should also have free access to outdoor areas [4,5]. It is a production system in which synthetic and chemical compounds are not used as feed components or to maintain the health of the chickens [1], and, importantly, it is environmentally friendly [6]. The existence of all these guidelines, related to the aspects of ecological chicken breeding, means that consumers are able to pay a higher price for products that have been produced naturally and present high nutritional value and quality [7,8].

Poultry meat production accounts for about 36% of the global meat production, and chicken meat accounts for about 89% of the total poultry production [5,9]. In 2020, the global production of poultry meat was over 136.5 million tons and increased by 2.4% compared to 2019 [9]. Poultry meat is characterized by its high quality, affordable price and short production cycles, and, most importantly, it is safe for consumers [10]. The quality of poultry products, including the content of nutrients and functional properties, is influenced by intensive selection, genotype, age, sex and type of production system [5,6,7,11,12]. In addition to the physicochemical and nutritional properties of meat, special attention has recently been paid to the histological traits of the pectoral muscles, also due to the increasing incidence of new myopathies in breast muscles, such as white striping, wooden breast and spaghetti meat [13,14,15,16,17,18,19]. Most of the available studies mainly investigated the effect of the genotype and management system on the quality of the chicken meat and fiber diameter without considering the difference between the sex of the birds. Sex, however, is a vital factor determining both the physicochemical parameters as well as the growth and slaughter performance of chickens [7]. In addition, the evident impact of sexual dimorphism on the size of carcasses directly affects other parameters of the slaughter performance of chickens and the quality of meat [6,7,20,21]. Usually, males have a higher final live body weight (BW), daily weight gain and carcass weight, which can affect and be related to other parameters [7,14]. Therefore, we decided to verify the hypothesis that sex has an influence on the growth performance, carcass traits and meat quality in organic Ross 308 broiler chickens and that this rapidly feathering breed will also prove useful in ecological production.

The content of fatty acids and their ratios are important criteria related to the health-promoting properties of meat [22]. Poultry meat is a good source of PUFA, especially n-3 PUFA, including eicosapentaenoic acid C20:5 n-3 and docosahexaenoic acid C22:6 n-3, which have a positive effect on the function of the brain and the cardiovascular system [23]. Other important criteria are, for example, the n-6/n-3 ratio of fatty acids and the atherogenic index (AI) and thrombogenic index (TI), and their values indicate a lower or higher risk of coronary heart disease or cancer [22,24]. Lower AI and TI values are positively correlated with a lower risk of serious abnormalities in the coronary arteries. In the human diet, the recommended values should be lower than 1 for AI and 0.5 for TI [25]. The sex of birds, in addition to their diet and genotype, is also an important parameter influencing the ratio and concentration of individual fatty acids [24,26,27].

The main aim of the experiment was to investigate the effect of sex on the growth performance, carcass traits and meat quality, as well as the fatty acid profile and histological parameters, of the pectoral muscles from organic broiler chickens.

## 2. Materials and Methods

### 2.1. Animals and Experimental Design

The experiment was conducted during the spring season, from March to May, in a poultry farm located in the central region of Poland (Kujawsko-Pomorskie Voivodeship). A total of 60 one-d-old Ross 308 chickens (half males and half females) were divided into 2 groups, according to the sex, and reared under organic conditions (Org.) till 82 d of age (10 birds, i.e., 5 males and 5 females in each 3 pens; replications for experimental groups). The area of the poultry house for each group was 3 m^2^ (3.3 birds/m^2^) with an outdoor yard of 13.3 m^2^/bird outdoor run availability. For the Org. group, the outdoor access from the pens was provided after 4 weeks of age during daylight hours (from 8:00 a.m. to 3:00 p.m.), and those chickens were exposed to natural environment (the average temperature was 13–15 °C). Birds were confined to indoor pens at night. The experiment was performed according to the Polish Local Ethical Commission (No. 22/2012) and in accordance with the animal welfare recommendations of European Union directive 86/609/EEC. During the whole time of the experiment, the birds’ health was under constant supervision. The birds were fed from the beginning with organic feed, consisting of 54% grains (wheat, triticale and oats), 30% legumes (pea, yellow lupine) and the remaining part press cake and rape oil, vitamins and fodder chalk and salt. Feed was prepared in an organic farm (certificate No. PL-EKO-07-04187, Łabiszyn, Poland) where the birds were kept. Basic chemical composition of the feed is presented in Table 1. All birds had unlimited access to water.

### 2.2. Slaughter Surveys

On the last day of rearing, all birds (males and females) were individually weighed (after a fasting period of 12 h) and transported (including careful catching and loading) to a commercial poultry slaughterhouse. After careful unloading and hanging in randomized order, all birds were electrically stunned and slaughtered. After evisceration, the hot carcass weight was recorded, and carcass yield was calculated. At slaughter, the pectoral muscle (PM) was removed from all carcasses and its percentage based on hot carcass weight was calculated. Afterward, it was vacuum packaged and stored frozen (−20 °C) until analyses.

### 2.3. Physicochemical Properties

The PM pH was measured using a portable pH-meter (pH-star Matthäus GmbH, Pöttmes, Germany) at 15 min (pH_15_) and 24 h (pH_u_) post mortem (according to Polish Standard PN-77/A-82058) [28]. Color measurements were performed at 24 h post mortem using the CIE system (L*, lightness; a*, redness; b*, yellowness) according to the method given by Litwińczuk et al. [29] using a spectrophotometer Shimadzu UV-1800 (Shimadzu Corporation, Tokyo, Japan).

The tenderness of PM was assessed using a multifunctional machine Instron 3342 (Instron Corporation, Norwood, MA, USA, 2005) with Bluehill Application for tensile tests with Warner–Bratzler shear device (Instron force transducer, Model 2519-104, Series No. 47452, Capacity 500N, S/N 47452), which allowed to register the maximum shear force at crosshead speed of 150 mm/min. Five cores (10 mm^2^ cross-sectional area and 50 mm length) were cut parallel to the muscle fibers, and each core was sheared 3 times. The average of 15 shears was expressed in N/cm.

Water-holding capacity (WHC) was measured on the right PM 24 h after slaughter. The sample was minced and analyzed by a method of Grau and Hamm [30] modified by Pohja and Ninivaara [31]. The measurement was performed using Whatman No. 1 filter paper. The obtained value was expressed as % hygroscopicity.

The protein content was calculated using Kjeldahl method, while the content of fat was determined by Soxhlet method [32].

The amount of total collagen was determined based on the content of hydroxyproline (conversion factor 7.52) according to PN-ISO3496:2000 [33]. Soluble collagen was determined using the method described by Palka [34]. The collagen solubility was calculated as the percentage of soluble collagen in the total collagen.

### 2.4. Histological Evaluation

From the carcasses, approximately 1 cm^3^ muscle sample of each PM was removed and immediately frozen in liquid nitrogen (−196 °C). Each specimen was cut in a cryostat (Cryostat Microm HM 525, GmbH, Germany, Thermo SCIENTIFIC, Series No. 52827, Runcorn, UK) into sections of 10 μm thick, which were then used for histochemical staining based on the hematoxylin and eosin method [35]. The microscopic images of the specimens (at the magnification of 200×) were taken using Opta-Tech microscope equipped with an Opta-View™ camera (Opta-Tech microscope, Warsaw, Poland), Model: MN-800, Series No. 04783. Histomorphometric analysis, including the calculation of the shortest diameters of 300 muscle fibers in each individual according to Brooke [36], was conducted by means of Multiscan 18.03 software for computer analysis of microscopic pictures (Computer Scanning Systems II Ltd., Warsaw, Poland).

### 2.5. Analysis of Fatty Acid Profile

The samples were subjected to lyophilization in a freeze-dryer (Lyovac GT2, Finn-Aqua, Tuusula, Finnland) and subsequently to homogenization using homogenizator (MPW-309, Warsaw, Poland) in an extraction mixture composed of chloroform and methanol in a 2:1 ratio in accordance with the method described by Folch et al. [37]. Next, fatty acid methyl esters were prepared. For this purpose, methylation of fatty acids with a 0.5 M solution of sodium methoxide was conducted. The samples were kept in an incubator at 37 °C for 22 h. Next, in order to extract fatty acid methyl esters, isooctane was introduced to the samples.

The analysis was performed using the 3800 GC type gas chromatograph with FID detector (Varian 3800 GC, Walnut Creek, CA, USA). The separation was conducted on the Supelcowax 10 GC column (dimensions 30 m × 0.25 mm × 0.25 µm) at a transfer temperature line of 230 °C, and, of the detector, 250 °C. The flow rate of the carrier gas (helium) was 1.5 (mL/min), and the volume of the injected sample was 1 µL. The fatty acid methyl esters were identified with Supelco PUFA-2 Animal Source and Supelco 37 Component Fame Mix standards (Supelco, Bellefonte, PA, USA). The composition of fatty acids was expressed as a percentage of the total fatty acids.

### 2.6. Measurement of Oxidative Stability (TBARS)

Prior to the analysis of oxidative stability, left breast muscle samples were kept in a freezer (−20 °C) for 3 mo. Lipid oxidation was determined by the thiobarbituric acid reactive substances (TBARS) method as described by Pikul et al. [38] on 10 g of raw meat after allowing it to thaw for 15 min. Briefly, 5.0 g of thawed meat was minced and homogenized using a 4% perchloric acid and alcohol solution of butylated hydroxytoluene (0.01%) at 9500 rev/min. After filtering with Whatman filter paper, the filtrates were diluted and washed with 4% perchloric acid and mixed. Next, a 0.02 M solution of TBA was added and the samples were heated (100 °C for 1 h). The absorbance at the wavelength of 532 nm was measured using a spectrophotometer UV-VIS (Shimadzu Corporation, Tokyo, Japan). The TBARS value was expressed as mg of malondialdehyde (MDA) per kilogram of raw meat using a standard curve prepared from 1,1,3,3-tetraethoxy-propane.

### 2.7. Statistical Analyses

The statistics meet the assumptions of normal distribution (which was verified using the Shapiro–Wilk test) and requirements for the homogeneity of variance, which are necessary for the use of parametric tests. The significance of differences between experimental groups were evaluated by the Student’s *t*-test. The values were given in terms of mean values and standard deviation (SD). The obtained data were processed using Statistica 13.1 software. Each bird formed the experimental unit.

## 3. Results and Discussion

### 3.1. Growth and Slaughter Performances

The growth and slaughter performances in the broiler chickens (males and females) are presented in Table 2.

As expected, the males were heavier (+5.7%) and the differences were significant (*p* < 0.05) and showed a higher carcass weight (+12.3%; *p* < 0.05) and higher carcass yield (+5.6%; *p* < 0.05), while the weight and yield of the PM were similar between the sexes (*p* > 0.05). Moreover, the daily weight gain (DWG) in males was significantly higher at *p* > 0.05. A similar trend was observed by van der Sluis [39] in ISA broiler chickens fed with organic feed and Tůmová et al. [20] for Ross, JA and Dual chickens. In traditional rearing systems (intensive vs. semi-intensive), the sex of the birds was a factor determining slaughter performance, as confirmed in studies by Cygan-Szczegielniak et al. [7]. Moreover, in this case, the males were characterized by a statistically significantly higher final live body weight (final BW), DWG and carcass and pectoral muscle weight [7]. A study by Maiorano et al. [40] also confirmed significantly higher values of final BW, carcass weight, carcass yield and pectoral muscle weight in male Ross chickens slaughtered at the age of 42 days. A clear sexual dimorphism in relation to body weight was also noted in Milanino hens managed in the free-range system [41]. The inherent differences in the size of carcasses between the sexes directly influence other parameters of the slaughter performance in chickens [6,7,20,21].

### 3.2. Physicochemical Traits and Fiber Diameter

The physicochemical traits and fiber diameter of the PM are reported in Table 3.

Sex did not influence (*p* > 0.05) the pH measured at 15 min and 24 h post mortem, as well as the color and WHC. A high pH of muscles usually results in a shorter shelf life of the meat, and the post mortem drop in pH is one of the most important processes necessary for the transformation of muscles into meat, which, in turn, directly influences the meat’s tenderness, color and WHC [42]. In our study, the sex of the birds had no effect on the WHC, which corresponded to the results obtained for the parameter directly related to it, i.e., the pH. Similar findings were reported by Połtowicz and Doktor [43], who studied the effect of the free-range raising system using the fast-growing Ross 308 line. The pHu value (5.63) is within the pH range accepted for commercial meats. A relatively low and stable pH is characteristic of meat from organic production systems where chickens have constant access to outdoor areas, which ensures their welfare and reduces pre-slaughter stress, thus reducing the amount of glycogen released in the muscles [5]. Consistent with our study, Goo et al. [21] reported that sex had no effect on meat quality traits such as the pH, WHC or meat color. Color has been considered an important indicator of meat quality driving consumer choices [5,44]. One factor that may affect the meat color is the poultry production system. In our study, the relatively high values of a* for chicken meat could be attributed to the physical activity of the birds and their access to outdoor areas. This trend was also observed by Galvez et al. [5] in the case of organic chickens. A higher level of physical activity promotes an increase in the content of myoglobin, which is directly related to a greater value of redness [5]. The higher yellowness (compared to that usually noted in intensive systems) of the meat (b*) measured in our own study could also be related to the access of chickens to outdoor areas and, thus, to a diet rich in plants containing a high amount of carotenoid pigments [8,45]. Consistent with our study, Fanatico et al. [46] found no differences in the value of b* between male and female chickens.

Statistically significant differences (*p* < 0.05) were found between the males and females reared under the organic system in regard to the dry matter (%) and protein (%) in the PM (Table 3). In both cases, the results were higher in the females, respectively, by 3.4% and 4.14%. In the meat of the females, statistically lower fat content was demonstrated (*p* < 0.05). Although the sex of the birds played a key role in this case, in general, the pectoral muscles of chickens, regardless of their sex, are characterized by a high content of protein and a low content of fat. The high level of physical activity in the chickens managed in the organic system, compared to the results reported by other authors for intensive production systems, could be a reason why myogenesis is favored instead of lipogenesis [5,8], and this explains the obtained results. All the parameters measured in this study for the physicochemical traits of the breast muscles did not differ from the values measured in the muscles from chickens managed in the same production system and reported by other authors [5,6]. Our study revealed that the sex of the birds was a factor determining the value of the analyzed traits, and, therefore, this aspect should be taken into consideration.

**Figure 1:**
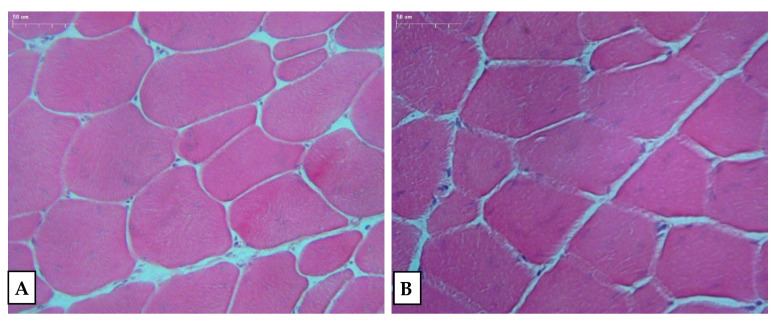
Microscopic picture of pectoral muscle of (**A**) male and (**B**) female organically reared Ross 308 broiler chickens, hematoxylin and eosin (H&E) staining, magnification: 200×.

The appearance and tenderness are two extremely important traits in poultry meat quality [47]. In particular, meat tenderness is the single most important sensory property affecting the final quality assessment [47], and it is an important attribute for consumers. Tenderness is affected by several factors, such as breed, sex, age, fiber resistance, sarcomere length, pH and collagen morphology [14,48,49,50]. In the present study, the meat from male chickens had a lower (*p* < 0.05) shear force value than that of female birds, indicating that the meat from the male birds is more tender. This could be due to the fiber diameter that was significantly smaller (*p* < 0.05) in the male chickens (Figure 1).

The smaller thickness of the fibers beneficially affects the meat quality and might be considered an indicator of fibrillarity and a delicate structure of the meat [40]. Berri et al. [51] also confirmed the significant effect of sex on most of the analyzed characteristics, including the fiber diameter. The greater diameter of the muscle fibers in the female birds was associated with a higher plasma creatine kinase activity, i.e., a factor influencing muscle growth, which differentiated both sexes [51]. The content of total collagen and soluble collagen and other parameters were similar (*p* > 0.05) between the sexes. The collagen content was lower in the female birds, but the differences were not statistically significant. A similar trend was noted by Tavaniello et al. [52] in quails, whose collagen content was significantly lower in females, and this could be related to differences in the hormone metabolism between male and female birds [52]. Collagen content is an important parameter influencing the hardness and quality of meat [45,52]. In our study, the low collagen content certainly had an effect on the measured high tenderness of the meat from the chickens managed in the organic system. As has been demonstrated for the aforementioned system, sex is one of the factors that may affect the meat traits in question. A great deal of data that would confirm this tendency for the organic system were observed. The research revealed that the crucial factors affecting most of the parameters under investigation were undoubtedly the sex and feeding system, which has also been proved by other authors [7,53].

### 3.3. Fatty Acid Profile and Oxidative Stability (TBARS)

Table 4 presents the fatty acids composition in broiler meat depending on the sex of the birds.

Considering saturated fatty acids (SFA), the highest concentration was found for C16:0, which was about 39% of the total SFA for both groups. With respect to monounsaturated acids (MUFA), the highest concentration was found for C18:1 n-9, and it ranged from 23.96 to 24.41% of the total MUFA in the females and males, respectively (Table 4). There were no statistically significant differences between the meat samples from male and female chickens in the content of this acid. The total concentration of PUFA was similar in the meat from female and male birds and accounted for approximately 19% of the total content of fatty acids. Considering polyunsaturated acids (PUFA), the highest concentration was measured for C18:2 n-6, and it was significantly higher (14.98%) in the meat from males, similar to the studies by Onk et al. [27] and Cerolini et al. for Milanino chickens [26]. An analysis of the meat from the broilers revealed the highest levels of n-3 fatty acids in the samples obtained from females. The levels of eicosatrienoic acid (C20:3 n-3), eicosapentaenoic acid (C20:5 n-3) and docosahexaenoic acid (C22:6 n-3) were significantly higher in females and accounted for 4.51, 0.336 and 0.683% of the total fatty acids, respectively, which is in line with the studies by Cerolini et al. [26]. Most of the identified PUFAs represented those from the n-3 group, which is characteristic of meat from organic production systems. These beneficial proportions can have a positive effect on the juiciness and tenderness of the meat [26,27]. The n-3/n-6 ratio is important due to the effect of these polyunsaturated fatty acids on health. Moreover, the values of AI and TI are crucial for the health of consumers. There were no statistically significant differences in the atherogenic and thrombogenic indices between the male and female birds. The AI values for the meat from both groups were within the recommended levels, but the TI was 3-fold higher than the recommended value. The analysis of the meat from the broilers revealed that the n-3/n-6 ratio of fatty acids was significantly higher in the samples obtained from females than in males (0.395 vs. 0.257%, respectively). On the other hand, the n-6/n-3 ratio was significantly higher in the males, and the values ranged between 3.23 for females and 4.76% for males, which was also consistent with the results of Cerolini et al. [26]. Because of the risk of coronary heart disease and cancer, the recommended values of the n-6/n-3 ratio should be lower than 4.0, and the ratio of PUFA to SFA higher than 0.4, which is very difficult to achieve in poultry and other meat products [22]. Considering the relationships identified in our study, i.e., a higher content of n-3 polyenoic acids, higher n-3/n-6 ratio and the n-6/n-3 ratio in females lower than 4.0, meat from female birds is more beneficial for consumer health.

The TBARS value was measured to verify the oxidative stability of the muscle tissue, and the results are presented in Table 4. The TBARS represents the level of lipid oxidation and is a value describing the content of malondialdehyde, ketones and similar oxidation products. There were no significant differences in the TBARS levels between the male and female birds. The oxidative stability was similar in both experimental groups and ranged from 0.672 to 0.681 mg MDA/kg of muscle. The slightly higher TBARS values obtained in the present study can be explained by the higher physical activity of the animals reared in systems with access to the outdoors, which translates into an increased content of total and heme Fe catalyzing peroxidation, and this, in turn, promotes the oxidative metabolism in muscles and, thus, increased lipid peroxidation [45]. Moreover, Funaro et al. [45] obtained a higher TBARS concentration in the chicken meat from the free-range system than from the conventional one. Research by other authors confirmed that the TBARS level is mostly influenced by the rearing system, physical activity and cooking of the meat [24,45].

Consumer preferences regarding poultry meat from the broilers managed in organic systems may concern not only factors related to the improved growth performance and meat quality but also those related strictly to animal welfare.

## 4. Conclusions

In conclusion, the results of the research confirm the hypothesis that sex is a factor influencing some of the characteristics related to the growth, carcass traits and quality of the meat of Ross 308 broiler chickens kept in an ecological system. They also prove the suitability of this breed for ecological production. Compared to the female birds, the male birds exhibited: (i) a lower fiber muscle diameter and shear force; (ii) a lower dry matter and protein; (iii) a meat with higher fat content. Sex had an effect on the performance and meat quality of the fast-growing chickens reared under the organic system: the males had a heavier carcass weight and yield, final BW and a higher DWG. Sex also had an effect on the fatty acid profile: the males had a lower content of n-3 polyenoic acids, lower n-3/n-6 and higher n-6/n-3 ratio. Moreover, the suitability of these fast-growing broilers for natural and organic production systems has been researched with regard to the performance, meat quality and the histological characteristics of the muscles. However, this part of the research should be supplemented with further analyses.

## Figures and Tables

**Table 1:** Chemical composition of feed supplied to chickens reared under organic (Org.) systems.

Rearing System	Org.
ME (kcal/kg)	2319.19
DM (%)	92.61
Ash (%)	4.85
CP (%)	16.71
Lipid (%)	3.28
Fiber (%)	5.44
NDF (%)	11.46
ADF (%)	5.58
ADL (%)	1.05

NDF-neutral detergent fiber; ADF-acid detergent fiber; ADL-acid detergent lignin.

**Table 2:** Effects of sex on growth and slaughter performance of chickens reared in the organic system at 82 days (mean values ± SD).

Item	Sex		
	Male	Female	*p*-Value
n	30	30	
Final live BW (g)	3048.0 ^a^ ± 44.80	2882.7 ^b^ ± 49.29	*p* = 0.019
Daily weight gain (g)	53.68 ^a^ ± 1.46	50.73 ^b^ ± 1.57	*p* = 0.047
Carcass weight (CW, g)	2368.0 ^a^ ± 31.46	2109.1 ^b^ ± 37.01	*p* = 0.037
Carcass yield (%)	77.52 ^a^ ± 1.21	73.4 ^b^ ± 1.42	*p* = 0.046
Pectoral muscle (g)	500.5 ± 11.04	475.0 ± 13.21	*p* = 0.529
Pectoral muscle (% CW)	21.10 ± 1.54	22.19 ± 1.36	*p* = 0.264

Significance: ^a,b^ *p* < 0.05; n-number of animals; SD-standard deviation.

**Table 3:** Effects of sex on physicochemical traits and fiber diameter of the pectoral muscle of chickens reared in the organic system at 82 days (mean values ± SD).

Item	Sex		
	Male	Female	*p*-Value
n	30	30	
pH_15_	6.02 ± 0.221	5.96 ± 0.207	*p* = 0.424
pH_24_	5.63 ± 0.063	5.63 ± 0.067	*p* = 0.956
Color 24 h			
L*	61.67 ± 2.42	60.58 ± 2.36	*p* = 0.222
a*	10.27 ± 1.21	10.18 ± 1.31	*p* = 0.848
b*	5.05 ± 0.97	5.83 ± 0.87	*p* = 0.069
WHC ^1^ (%)	62.83 ± 2.58	63.54 ± 2.32	*p* = 0.517
Shear force (N/cm)	35.45 ^a^ ± 1.97	37.45 ^b^ ± 1.66	*p* = 0.035
Fiber diameter (μm)	41.42 ^a^ ± 1.69	42.79 ^b^ ± 1.91	*p* = 0.046
Dry matter (%)	23.98 ^a^ ± 1.10	24.80 ^b^ ± 0.998	*p* = 0.042
Protein (%)	22.94 ^a^ ± 1.27	23.89 ^b^ ± 0.891	*p* = 0.025
Fat (%)	1.47 ^a^ ± 0.078	1.14 ^b^ ± 0.058	*p* = 0.041
Total collagen (%)	0.51 ± 0.051	0.46 ± 0.046	*p* = 0.590
Soluble collagen (%)	0.16 ± 0.012	0.14 ± 0.015	*p* = 0.571

Significance: ^a,b^ *p* < 0.05; ^1^ WHC-water holding capacity; n-number of animals; SD-standard deviation.

**Table 4:** Effect of sex on the content of fatty acids (% of total acids) and oxidative stability (mg MDA/kg) in pectoral muscles from broilers reared in an organic system at 82 days (mean values ± SD).

Item	Sex		
	Male	Female	*p*-Value
n	30	30	
C14:0	0.764 ^a^ ± 0.112	0.603 ^b^ ± 0.031	*p* = 0.012
C16:0	39.09 ± 0.411	39.17 ± 0.514	*p* = 0.990
C18:0	12.52 ± 0.29	13.34 ± 0.451	*p* = 0.143
C20:0	1.55 ^a^ ± 0.101	1.17 ^b^ ± 0.091	*p* = 0.009
C22:0	0.443 ^a^ ± 0.049	0.559 ^b^ ± 0.047	*p* = 0.038
∑ SFA	54.36 ± 0.321	54.77 ± 0.308	*p* = 0.373
C16:1	1.591 ^a^ ±0.104	1.254 ^b^ ± 0.116	*p* = 0.042
C18:1 n-9	24.41 ± 0.544	23.96 ± 0.616	*p* = 0.595
C20:1	0.474 ^a^ ± 0.02	0.387 ^b^ ± 0.019	*p* = 0.014
∑ MUFA	26.47 ± 0.63	25.61 ± 0.709	*p* = 0.372
C18:2 n-6	14.98 ^a^ ± 0.329	13.85 ^b^ ± 0.235	*p* = 0.008
C20:2 n-6	0.252 ± 0.016	0.247 ± 0.019	*p* = 0.859
C20:3 n-3	3.20 ^a^ ±0.413	4.51 ^b^ ± 0.628	*p* = 0.049
C20:5 n-3	0.249 ^a^ ± 0.031	0.336 ^b^ ± 0.032	*p* = 0.046
C22:6 n-3	0.467 ^a^ ± 0.064	0.683 ^b^ ± 0.097	*p* = 0.041
∑ PUFA	19.15 ± 0.657	19.62 ± 0.767	*p* = 0.653
PUFA/SFA	0.353 ± 0.012	0.358 ± 0.015	*p* = 0.766
∑ n-3	3.92 ^a^ ± 0.501	5.52 ^b^ ± 0.743	*p* = 0.018
∑ n-6	15.23 ^a^ ± 0.338	14.09 ^b^ ± 0.249	*p* = 0.011
n-3/n-6	0.257 ^a^ ± 0.033	0.395 ^b^ ± 0.055	*p* = 0.045
n-6/n-3	4.76 ^a^ ± 0.616	3.23 ^b^ ± 0.421	*p* = 0.047
AI	0.925 ± 0.017	0.919 ± 0.017	*p* = 0.812
TI	1.62 ± 0.063	1.49 ± 0.071	*p* = 0.176
TBARS	0.672 ±0.071	0.681 ± 0.09	*p* = 0.789

Significance: ^a,b^ *p* < 0.05; AI-Atherogenic index; TI-Thrombogenic index; TBARS-oxidative stability; n-number of animals; SD-standard deviation.

## Data Availability

The data presented in this study are available on request from the corresponding author.
